# The Slaughterhouse as Hotspot of CC1 and CC6 *Listeria monocytogenes* Strains with Hypervirulent Profiles in an Integrated Poultry Chain of Italy

**DOI:** 10.3390/microorganisms11061543

**Published:** 2023-06-09

**Authors:** Fabrizia Guidi, Gabriella Centorotola, Alexandra Chiaverini, Luigi Iannetti, Maria Schirone, Pierina Visciano, Alessandra Cornacchia, Silvia Scattolini, Francesco Pomilio, Nicola D’Alterio, Marina Torresi

**Affiliations:** 1 Istituto Zooprofilattico Sperimentale Dell’abruzzo e del Molise “G. Caporale”, Via Campo Boario, 64100 Teramo, Italy; f.guidi@izs.it (F.G.); a.chiaverini@izs.it (A.C.); l.iannetti@izs.it (L.I.); a.cornacchia@izs.it (A.C.); s.scattolini@izs.it (S.S.); n.dalterio@izs.it (N.D.); m.torresi@izs.it (M.T.); 2 Faculty of Bioscience and Technology for Food, Agriculture and Environment, University of Teramo, Via R. Balzarini, 1, 64100 Teramo, Italy; mschirone@unite.it (M.S.); pvisciano@unite.it (P.V.)

**Keywords:** *Listeria monocytogenes*, whole genome sequencing, poultry, hypervirulent profiles, persistence, slaughterhouse

## Abstract

In Europe, very few studies are available regarding the diversity of *Listeria monocytogenes (L. monocytogenes)* clonal complexes (CCs) and sequence types (ST) in poultry and on the related typing of isolates using whole genome sequencing (WGS). In this study, we used a WGS approach to type 122 *L. monocytogenes* strains isolated from chicken neck skin samples collected in two different slaughterhouses of an integrated Italian poultry company. The studied strains were classified into five CCs: CC1-ST1 (21.3%), CC6-ST6 (22.9%), CC9-ST9 (44.2%), CC121-ST121 (10.6%) and CC193-ST193 (0.8%). CC1 and CC6 strains presented a virulence gene profile composed of 60 virulence genes and including the Listeria Pathogenicity Island 3, *aut_IVb*, *gltA* and *gltB*. According to cgMLST and SNPs analysis, long-term persistent clusters belonging to CC1 and CC6 were found in one of the two slaughterhouses. The reasons mediating the persistence of these CCs (up to 20 months) remain to be elucidated, and may involve the presence and the expression of stress response and environmental adaptation genes including heavy metals resistance genes (*cadAC, arsBC, CsoR-copA-copZ*), multidrug efflux pumps (*mrpABCEF, EmrB, mepA, bmrA, bmr3, norm*), cold-shock tolerance (*cspD*) and biofilm-formation determinants (*lmo0673, lmo2504, luxS, recO*). These findings indicated a serious risk of poultry finished products contamination with hypervirulent *L. monocytogenes* clones and raised concern for the consumer health. In addition to the AMR genes *norB, mprF, lin* and *fosX*, ubiquitous in *L. monocytogenes* strains, we also identified *parC* for quinolones, *msrA* for macrolides and *tetA* for tetracyclines. Although the phenotypical expression of these AMR genes was not tested, none of them is known to confer resistance to the primary antibiotics used to treat listeriosis The obtained results increase the data on the *L. monocytogenes* clones circulating in Italy and in particular in the poultry chain.

## 1. Introduction

The European Union (EU) is one of the world’s largest poultry meat producers and a net exporter of poultry products with annual production of around 13.4 million tons [1]. The EU agricultural outlook for 2021–2031 estimated a pro capite increase from 23.5 kg in 2021 to 24.8 kg in 2031 for the poultry sector. This should be managed through a healthier image of poultry relative to other meats, greater convenience in its preparation and the absence of religious constraints in its consumption.

*L. monocytogenes* is a ubiquitous bacterium causing human listeriosis, the most serious foodborne disease under EU surveillance with the highest proportion of hospitalized cases (96.5% in 2021) and fatality rate (13.7% in 2021). Invasive forms of the disease mainly affect people at risk causing abortion and stillbirth in pregnant women and meningitis septicemia and death in the elderly, immunocompromised people, and new-borns [2].

Listeriosis associated with meat products is a significant food safety concern and slaughterhouses and market sites are ideal environments for the proliferation of *L. monocytogenes* [3]. Although the occurrence of *L. monocytogenes* in meat products is the highest in products of bovine or porcine origin [2,4], it is also present in chicken and other poultry meats, including packaged and non-packaged products, whole chickens and various types of sliced meat. Contamination of chicken meat and foods prepared using chicken meat can occur at various stages before marketing, including the primary production stage, abattoir, processing plant, and retail stores [5]. In recent years, numerous studies on the presence of *L. monocytogenes* in these matrices have been conducted worldwide [5,6,7,8,9]. The consumption of cooked chicken products was previously associated with the transmission of listeriosis and caused several listeriosis outbreaks in different countries [10,11,12,13].

Some *L. monocytogenes* strains are able to survive and persist in food-producing environments (FPE) even for years, due to the adaptation to different environmental stresses, resistance to disinfectants and biofilm formation. In a FPE, hard to reach surfaces during clean-up and sanitation could be ideal niches for *L. monocytogenes* persistence and act as persistent source of contamination [14,15,16,17,18].

Currently, the whole genome sequencing (WGS) offers the highest level of discrimination for surveillance of pathogens and for epidemiological investigations of foodborne outbreaks, also allowing the characterization of pathogenic microorganisms. WGS is considered to be the best approach to obtain the most detailed results on the nature and localization of genes associated with virulence, biological fitness and antimicrobial resistance [19].

WGS data on *L. monocytogenes* in poultry samples are reported worldwide, in particular are available data from poultry meat in China, from chicken in South Korea and from turkey in USA [9,20,21]. On the contrary, at European level, particularly in Italy, very few studies regarding the spread of *L. monocytogenes* clonal complexes (CCs) in poultry and the related genomic typing of isolates using WGS are available. More data on the genetic virulence profile is needed in order to precisely determine the pathogenic potential of *L. monocytogenes* isolates and to acquire a better comprehension of the risk exposure for the consumers. Moreover, the investigation of genetic traits for stress resistance and tolerance to disinfectants and heavy metals is important to understand the ability of the strains to adapt and persist in the production environment. The latter aspect is becoming relevant in intensive poultry production which increases the risk of microbial contamination and promotes the persistence of food-borne pathogens in environmental niches. It is well known that certain CCs, such as CC1, CC2, CC4, and CC6, are more frequently associated with clinical cases and are hypervirulent in a humanised mouse model, whereas others like CC9 and CC121 are mainly of foodborne origin and show hypovirulence in vivo [14,22,23,24,25]. Different virulence profiles have been observed between hyper- and hypovirulent clones of *Listeria monocytogenes* with the former carrying additional virulence determinants such as Listeria Pathogenicity Islands (LIPIs) in addition to the ubiquitous LIPI-1 [22,26,27]. Conversely, hypovirulent clones are better adapted to food processing environments, with a higher prevalence of stress resistance and benzalkonium chloride tolerance genes [22,27].

In this study, we used a WGS approach to type 122 *L. monocytogenes* strains isolated from poultry neck skin samples collected in two different slaughterhouses of an integrated Italian poultry company during a previous work performed by Iannetti et al. [7]. These authors used pulsed field gel electrophoresis (PFGE) to type strains and identify the persistent pulsotypes.

Specifically, in the present study we carried out WGS data analysis: (i) to identify the main *L. monocytogenes* CCs circulating in two poultry slaughterhouses; (ii) assess the clustering of the strains evaluating their persistence over time, (iii) determine the virulence genetic profiles of the isolates identifying hypervirulent clones and (iv) detect genes involved in stress response and tolerance to heavy metals and disinfectants.

## 2. Materials and Methods

### 2.1. Strain Collection

The strains collection derived from a previous study in which prevalence of *L. monocytogenes* in poultry along an Italian production chain was evaluated [7]. A total of 2080 samples (1560 faeces/caeca contents and 520 neck skin samples) from broiler chickens were analysed for *L. monocytogenes* detection. The broiler chickens were bred in farms of Northern and Central Italy and slaughtered in two different slaughterhouses of Central Italy, in particular M1 (first slaughterhouse) and M2 (second slaughterhouse).

The overall positivity of the collected samples was 6.63%, with 138 positive samples out of 2080. No positivity was recorded in samples of faeces, while an overall prevalence of 26.7% was detected in neck skin samples collected in the two slaughterhouses.

In this study 122 isolates (71 collected at M1 and 51 at M2), derived from positive neck skin samples, were typed using WGS and different bioinformatics analysis (Supplementary Table S1).

### 2.2. Whole Genome Sequencing (WGS) Analysis

DNA extraction was performed using QIAamp DNA Mini Kit (Qiagen Hilden, Germany) according to the manufacturer’s protocol with minor modifications according to Portmann et al. [28]. DNA quantity and quality were evaluated with Qubit fluorometer (Thermo Fisher Scientific, Waltham, Massachusetts, USA) and Eppendorf BioSpectrometer fluorescence (Eppendorf s.r.l., Milano, Italy). DNA integrity was assessed with Agilent 4200 TapeStation system (Agilent, Santa Clara, CA, USA).

Starting from 1 ng of input DNA, the Nextera XT DNA chemistry (Illumina, San Diego, CA) for library preparation was used according to the manufacturer’s protocols. WGS was performed on the NextSeq 500 platform (Illumina, San Diego, CA, USA) with the NextSeq 500/550 mid output reagent cartridge v2 (300 cycles, standard 150-bp paired-end reads).

For the analysis of WGS data, an in-house pipeline [29] was used which included steps for trimming (Trimmomatic v0.36) [30] and quality control check of the reads (FastQC v0.11.5) [31]. Genome *de novo* assembly of paired-end reads was performed using SPAdes v3.11.1 [32] with default parameters for the Illumina platform 2 × 150 chemistry (–only-assembler –careful –k21, 33, 55, 77). Subsequently, the genome assembly quality check was performed with QUAST v.4.3 [33].

All the genomes that met the quality parameters recommended by Timme et al. [34], such as average read quality Q score for R1 and R2 ≥ 30; Average coverage ≥ 20, number of contigs ≤ 300, and were used for the analysis.

The genome assemblies were deposited at DDBJ/ENA/GenBank under the BioProject PRJNA947067 (Supplementary Table S1).

### 2.3. Multilocus Sequence Typing, Core Genome Multilocus Sequence Typing and Single-Nucleotide Polymorphism Analysis

A multilocus sequence typing (MLST) analysis was performed by deducing the sequence type (ST) and the CC in silico, using the specific tool available from the BIGSdb-*Lm* database (https://bigsdb.pasteur.fr/listeria/; accessed on 20 February 2023) [35]. The MLST scheme used, included the seven housekeeping genes *abcZ*, *bglA*, *cat*, *dapE*, *dat*, *ldh*, and *lhlA* [35].

To verify the relatedness among the isolates, identifying genomic clusters, a core genome MLST (cgMLST) analysis was performed using the chewBBACA allele calling algorithm [36] and the Pasteur Institute cgMLST scheme of 1748 loci [37]. According to the guidelines for *L. monocytogenes* cgMLST typing [37], only the genomes with at least 1660 called loci (95% of the full scheme) were considered. GrapeTree [38] was used for the visualization of the minimum spanning tree (MSTreeV2 method).

A core single-nucleotide polymorphism (SNPs) analysis was performed using the CFSAN pipeline [39], and the sequence reads were mapped against an EGD-e reference genome (NC_003210.1). The resulting maximum likelihood (ML) tree was visualized using Interactive Tree of Life (iTOL) (https://itol.embl.de/; accessed on 20 February 2023).

The threshold values adopted in this study for cluster definition were an allelic distance (AD) of 7 for the cgMLST analysis [37] and 21 pairwise SNPs [40,41].

In order to find clones matching with strains belonging to the national collection of the Italian National Reference Laboratory for *L. monocytogenes* (NRL-*Lm*), the relative genome database (about 5000 *L. monocytogenes* genomes stored from food, environment and animals) was interrogated. In particular, a cgMLST analysis was performed comparing the genomes of the isolates of the study with all the genomes belonging to the same CC collected in the Italian database.

### 2.4. Genetic Determinants Involved in Virulence Potential, Antimicrobial Resistance, Stress Adaptation and Heavy Metal and Disinfectant Resistance

*In silico* analysis was performed using tools built in the BIGSdb-*Lm* platform for the detection of virulence genes, antimicrobial resistance (AMR) genes, heavy metal and disinfectants resistance determinants, stress survival islets (SSIs) and biofilm-associated genes.

The detection of additional determinants, not included in these schemes, was performed automatically using Prokka v.1.1211 [42].

## 3. Results

### 3.1. MLST Analysis

All the 122 genomes met the quality parameters recommended, such as average coverage ≥ 20X, *de novo* assembly length 2.7–3.2 Mbp and number of contings ≤ 300, and were used for the analysis.

**Figure 1:**
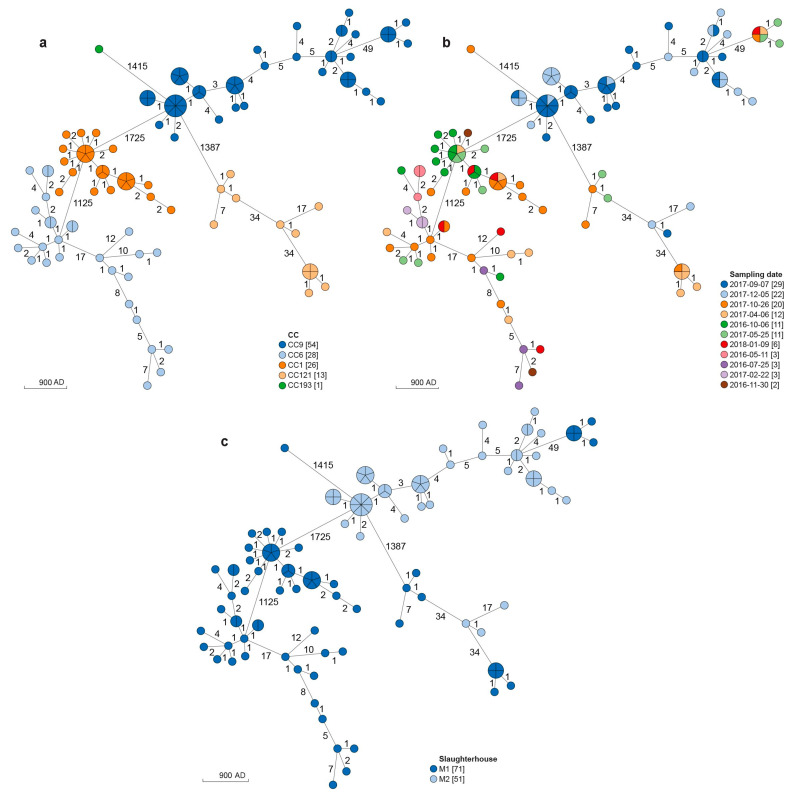
Cluster analysis of *L. monocytogenes* strains based on cgMLST profiles. (**a**) In the minimum spanning tree (MSTv2), strains are colored according to the Clonal Complex (CC). (**b**) In the MSTv2, strains are colored according to the sampling date. (**c**) In the MSTv2 strains are colored according to the slaughterhouse from which they were isolated. The scale bar indicates the allelic distance (AD).

The 122 isolates from M1 (n = 71) and M2 (M = 51) were classified into 5 STs and as many CCs (Table 1; Figure 1a; Supplementary Table S1): CC1-ST1 (26 strains; 21.3%), CC6-ST6 (28 strains; 22.9%), CC9-ST9 (54 strains; 44.2%), CC121-ST121 (13 strains; 10.6%) and CC193-ST193 (1 strain; 0.8%). Among the strains isolated from neck skin samples collected in M1, 28 belonged to CC6 (39.4%), 26 to CC1 (36.6%), 10 to CC121 (14.1%), 6 to CC9 (8.5%) and one strain belonged to CC193. Strains from M2 belonged to CC9 (48 strains; 94.1%) and CC121 (3 strains; 5.9%).

### 3.2. cgMLST and SNPs Analyses

**Figure 2:**
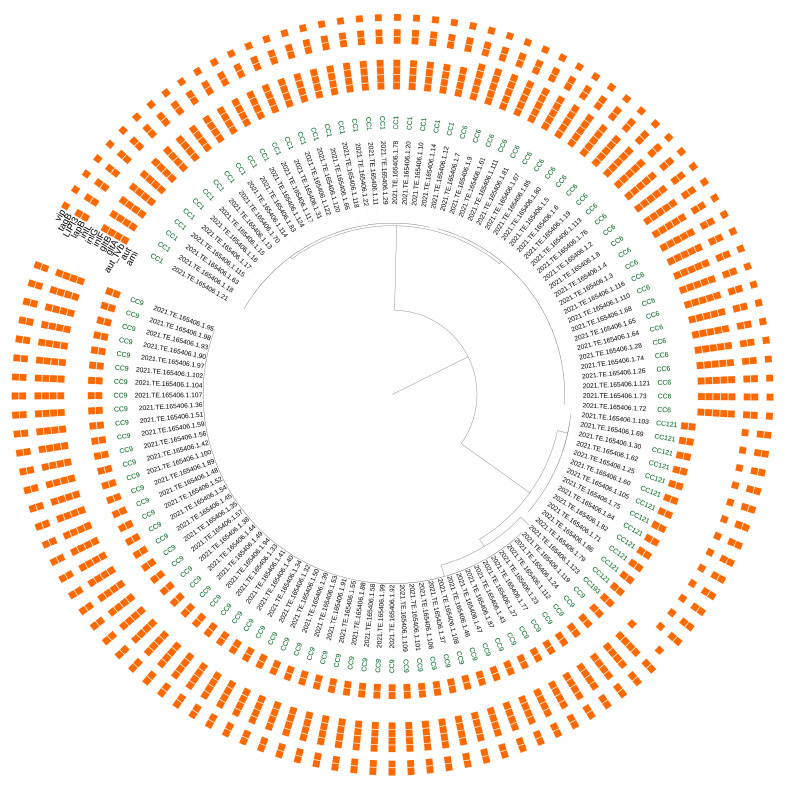
SNPs analysis of *L. monocytogenes* strains. The first circle indicates the CC; the presence/absence matrix represents virulence genes that vary between CCs.

The cluster analysis of the studied strains was performed using the results from both cgMLST and SNPs analysis (Figure 1 and Figure 2).

According to the minimum spanning tree (MST) obtained from cgMLST analysis (Figure 1a–c) and the SNPs distance matrix (Supplementary Table S2), all the CC1 strains grouped into a single cluster isolated in the M1 slaughterhouse from October 2016 to January 2018 (AD ranging from 0 to 7; SNPs of difference ranging from 4 to 10) (Table 2; Figure 1b,c).

Strains belonging to CC6 grouped into five main clusters. The largest CC6 cluster (16 strains; 0–7 AD; 1–17 SNPs) was isolated from May 2016 to January 2018, the second largest one (4 strains; 7 AD; 6–12 SNPs) from July 2016 to January 2018 and the third one (3 strains; 1 AD; 1–3 SNPs) from July 2016 to January 2018. One of the two smallest clusters was isolated from April to October 2017 (2 strains; 1 AD; 0 SNPs) and the other one included two strains both isolated at April 2017 (1 AD; 2 SNPs) (Table 2; Figure 1b,c).

Strains belonging to CC9 showed four main clusters. Three of them, all isolated in M2, were highly related and the remaining one, isolated in M1, was more distant. Among the M2 clusters, the largest one (30 strains; AD ranging from 0 to 7; 0–16 SNPs) and the second largest one (15 strains; AD ranging from 0 to 5; 1–20 SNPs) were both isolated from September to December 2017, while the third one (2 strains; a single AD and 0 SNPs of difference) was isolated in September 2017. The remaining CC9 cluster (6 strains; 0–1 AD; 1–6 SNPs) was isolated from samples collected from April 2017 to January 2018 (Table 2; Figure 1b,c).

The CC121 strains grouped into three main clusters. Two clusters were isolated in M1 from May to October 2017 (4 strains; 0–7 AD; 1–19 SNPs) and from April to October 2017 (6 strains; 0–1 AD; 0–5 SNPs) respectively. The remaining one (2 strains; a single AD and 1 SNP of difference) was isolated in M2 from September to December 2017 (Table 2; Figure 1b,c).

None of the 122 strains had a genetic correlation with those present in the NRL-*Lm* genome database. The cgMLST analysis showed that no distances ≤ 7 AD were found comparing the strains of this study with all the isolates of the same CC stored in the database.

### 3.3. Genetic Determinants Involved in Virulence Potential, Antimicrobial Resistance, Stress Adaptation and Heavy Metal and Disinfectant Resistance

Using the BIGSdb-*Lm* platform, 71 virulence genes were detected on a scheme of 93 targets. Fifty-two genes were carried by all the strains. Among these ubiquitous targets there were the conventional Listeria Pathogenicity Island (LIPI) 1, 9 internalins genes including *inlA*, *inlB*, *inlC*, *inlC2*, *inlD*, *inlE*, *inlH*, *inlJ* and *inlK* and the *virR*/*virS* virulence regulatory system. The variable genes showing differences in their presence/absence among different CCs were 19 (Figure 2). Overall, the CC1 and CC6 strains presented 66 virulence genes, while CC9, CC121 and CC193 carried 60, 57 and 56 virulence determinants, respectively.

More in detail, all the CC1 and the CC6 strains presented the additional LIPI-3, the teichoic acid biosynthesis genes *gltA* and *gltB*, and the invasion gene *aut_IVb*. The *lapB* gene was present in all the CCs except the CC193 strain. The virulence genes *ami* and *tagB* were carried by all the isolates belonging to CC9, CC121 and CC193. All the CC9 strains carried the Internalins genes *inlF*, also detected in the only CC193 strain, *inlG* and *inlL*.

The CC9, CC121 and CC193 strains showed different mutations in the *inlA* gene leading to a premature stop codon (PMSC). In particular, five PMSC mutation types were detected: PMSC_6 (all CC121 strains), PMSC_8 (48 CC9 strains), PMSC_25 (CC193) and PMSC_29 (6 CC9 strains). All the CC1 and CC6 strains carried a full length *inlA*.

All the tested strains presented the same AMR gene pattern including *fos* (fosfomycin), *lmo0919* (lincosamide), *lmo1695* (cationic antimicrobials), *norB* (fluoroquinolones) and *sul* (sulfanilamides). The additional AMR genes, *parC* for quinolones; *msrA* for macrolides and *tetA* for tetracyclines were also detected in all the strains. In addition to these AMR genes, different multidrug efflux pumps (*emrB*, *bmrA*, *bmr3*, *norM*) were detected in all the isolates.

The genetic determinants for different heavy metal resistance, multidrug efflux pumps and response to environmental stresses were reported in Table 3.

In particular, all the CC9 strains carried a complete SSI-1, while in all the CC121 a complete SSI-2 was found. Moreover, all the CC121 strains presented the Tn6188 transposon carrying the *qacH* gene which can confer reduced susceptibility to both quaternary ammonium compounds (QACs) and ethidium bromide. Only CC1 strains carried *cadA* and *cadC* for cadmium resistance.

Among the genetic determinants for arsenic resistance, *arsB* and *arsC* were found in all the studied strains while *arsA* and *arsD* were detected only in CC9 isolates. The *csoR-copA-copZ* copper resistance operon was present in all the strains while *copB* was only carried by 35 strains (34 CC9 and the CC193 isolate). All the isolates carried the cold-shock protein gene *cspD* and the genetic markers for biofilm production *lmo0673*, *lmo2504*, *luxS* and *recO.*

## 4. Discussion

Very few data are available at the European level on *L. monocytogenes* CCs and WGS analysis in poultry. Most of the existing studies have used old typing techniques such as serotyping and PFGE [5,6,43,44].

In this study 122 *L. monocytogenes* strains, isolated from two slaughterhouses of one of the main integrated Italian poultry companies during a previous study by Iannetti et al. (2020) [7], were typed using a WGS approach.

Ianneti et al., [7] used serotyping and PFGE to type the *L. monocytogenes* isolates identifying 18 different pulsotypes grouping into 8 main clusters. They also assumed the persistence of the pathogen since they isolated undistinguishable pulsotypes in carcasses slaughtered in the same slaughterhouse after periods up to 18 months long.

The WGS approach used in this study, allowed to better discriminate the strains and to obtain information on their genomes such as their virulence profile, the presence of AMR genes and of several determinants involved in environmental stress adaptation. Specifically, the MSTree, obtained from the cgMLST analysis, showed 78 different allelic profiles, grouping into 12 clusters and 6 singleton strains. The results obtained confirmed the long-term persistence of the pathogen in one of the slaughterhouse identifying clusters persisting up to 20 months.

As concluded by Iannetti et al. (2020) [7], considering the high number of carcasses processed in these slaughterhouses, it is probable that the environmental contamination derived from the sporadic introduction of *L. monocytogenes* strains through faeces of healthy carrier chickens and that once introduced, *L. monocytogenes* was able to persist in favourable niches thanks to its adaptation and surviving skills. Therefore, the slaughterhouse could be considered as an important *hotspot* of microbial contamination giving information on *L. monocytogenes* strains circulating at different point of the poultry chain.

In this study, we reported CCs already found by other authors worldwide in poultry and in particular CC1, CC6, CC9, CC121 and CC193. However, our results increased the data currently available at European and Italian level. Previous studies, mostly performed in China, reported the isolation of the following CCs from poultry meat: CC2, CC3, CC5, CC8, CC9, CC11, CC14, CC19, CC87, CC101, CC121, CC155, CC193 and CC307 [3,9,45,46]. Brown et al. [21] reported the isolation of a wide CCs diversity from the environment of turkey processing plants in the United States (CC1, CC2, CC3, CC5, CC6, CC7, CC8, CC9, CC11, CC29, CC87, CC89, CC155, CC224, CC321, CC506, CC554, CC570, CC1084) and the identification of persistent clones. Lee and Ruy reported the isolation of a CC1 strain from a chicken in South Korea [20].

Among the CCs found in this study, CC9 and CC121 have been previously defined hypovirulent clones, with a better adaptation to food processing environments. On the contrary, previous studies defined CC1 and CC6 as hypervirulent clones since they resulted most commonly associated with human listeriosis [14,22,23,24,25,47,48,49,50,51].

The microbial population associated with M1 was more heterogeneous than that isolated in M2. The results of cgMLST and SNPs analysis showed the presence of several clusters and none of them included strains isolated from both M1 and M2, indicating that each slaughterhouse presented an associated and unique microbial population. In M1, longer persistent clusters were detected (up to 20 months). Very interestingly, in this slaughter environment, CC1 and CC6 persisted for longer than CC9 and CC121, although the latter are commonly considered better adapted to food processing environments with a higher prevalence of stress resistance genes and a higher survival and biofilm formation [14,27].

All the genetic clusters detected in M2, belonging to CC9 and CC121, included strains isolated from September to December 2017 and so persisted for a shorter period.

Other authors also reported similar results [21,27,52,53] with hypervirulent clones such as CC1, CC2 and CC6 persisting for long time. These findings could indicate that the fitness of a strain is relative to the environment with which it is interacting. Therefore, within M1 may have been a selection pressure that favored CC1 and CC6 clones rather than the others. Moreover, besides the specific characteristics of the FPE (presence of ecological niches, non-compliant structures and equipment) and the survival abilities of the strains, other factors can influence *L. monocytogenes* persistence such as reintroduction of contaminated raw materials, inappropriate processing and ineffective cleaning and sanitizing protocols [27]. A limitation of this study was the lack of an intensive environmental sampling with multiple sampling sessions in the two slaughterhouses, in order to detect other persistent clones and to identify the surfaces acting as niches of persistence for *L. monocytogenes*. This should be useful to apply effective mitigation strategies. Another limitation was the lack of a genome comparison between the hypervirulent clones isolated in this study and those collected in the Italian genome database of *L. monocytogenes* clinical strains.

From the evaluation of the virulence profiles, it was quite evident that CC1 and CC6 isolates presented a higher number of virulence genes if compared with other CCs. In particular, all the CC1 and CC6 isolates presented both LIPI-1 and also the additional LIPI-3 known to confer a greater virulence to *L. monocytogenes* [54,55]. LIPI-1 carries the major virulence genes, such as *prfA*, the genes needed to escape from vacuoles (*hly* and *plcA*), genes for actin-based motility (*actA*) and genes needed for cell-to-cell spread (*mpl* and *plcB*). The LIPI-3 encodes the bacteriocin LLS, highly expressed in the intestine to alter host intestinal microbiota and allowing *L. monocytogenes* colonization of the intestine [56,57,58].

The *aut_IVb*, *gltA*, and *gltB* genes were also important virulence markers involved in invasion and teichoic acid biosynthesis respectively. These genes are typically carried by hypervirulent isolates and are absent in other *L. monocytogenes* leading a higher virulence potential [14,22,37].

All CC9, CC121 and CC193 strains did not present any LIPI other than the ubiquitous LIPI-1, and they harboured different types of PMSCs within the *inlA* gene [23,59,60] which are associated with attenuated virulence in mammal hosts [24,61,62].

All the strains isolated from M1 and M2 carried several determinants for stress adaptation, heavy metal resistance and tolerance to biocides. The prevalence of environmental adaptation determinants is commonly higher in hypovirulent CCs such as CC9 and CC121 [14,27,63]. However, in this study, many genes were also found in hypervirulent strains with some even exclusive to them, as in the case of *cadA* and *cadC*, only carried by CC1 strains. Genetic elements for arsenic and copper resistance [64,65], acid tolerance [66,67,68] and alkali response [66,69,70,71] were found in all the CCs as well as several multidrug efflux pumps, known to be involved in non-specific tolerance to disinfectants [26,72,73,74,75] (Table 1). The same result was obtained for the biofilm production markers *lmo0673, lmo2504, luxS* and *recO* [76] and for *cspD,* known to be essential for adaptation against various food-relevant stress conditions including cold growth [77]. Certain genetic determinants for environmental adaptation such as *arsAD*, *copB*, *inlL* and SSI-1 were only found in CC9 strains. The SSI-2 [78], and the Tn6188_qac gene were detected only in CC121 strains [79,80]. The presence of PMSCs mutations in the *inlA* gene, was previously found to be associated with increased biofilm production and was detected in CC9, CC121 and CC193 isolates [62]. However, further studies in this field are needed to identify factors that predispose some strains of *L. monocytogenes* towards increased biofilm formation [62].

In CC1 and CC6 strains, the presence of genetic determinants involved in stress response, such as heavy metal resistance, alkali and acid response and cold tolerance, may have contributed to the long-term persistence of these hypervirulent CCs in the M1 slaughterhouse. In the future, it might be interesting to evaluate the ability of these hypervirulent clones to produce biofilm *in vitro*.

The ability of *L. monocytogenes* strains to persist in food processing environments once introduced throughout the raw materials, together with the tendency of poultry slaughterhouses to act as contamination hotspots, could contribute to these food-associated environments becoming reservoirs of different *L. monocytogenes* genetic variants, including hypervirulent clones with adaptation skills [21].

The AMR genetic profile was the same in all the strains studied and included genes with mechanisms of antibiotic efflux (*norB*), antibiotic target alteration (*mprF*), and antibiotic inactivation (*lin, fosX*) [81,82,83,84]. Previous studies showed *fosX* and *lin* to be present in nearly all *L. monocytogenes* isolates [82,85]. This can be explained by native resistance to fosfomycin and lincosamides reported in *L. monocytogenes* strains. In addition to these ubiquitous AMR genes, we identified, in all tested strains, *parC* for quinolones, *msrA* for macrolides and *tetA* for tetracyclines, which were recently reported in *L. monocytogenes* by other authors [81,86]. Although the phenotypical expression of these AMR genes was not tested, none of them is known to confer resistance to the primary antibiotics used to treat listeriosis [85].

## 5. Conclusions

This study deepens the current knowledge on *L. monocytogenes* CCs circulating in the Italian poultry chain and on their genetic features, using WGS data analysis. Useful information was provided to integrate the limited data available in this regard at European level and in particular in Italy.

Among the detected CCs, CC1 and CC6, known to be hypervirulence clones, were found to persist for long time in the slaughterhouse.

These findings indicate a serious risk of finished food contamination with hypervirulent *L. monocytogenes* clones and raise concern for the consumer health.

As a future perspective, it would be interesting to evaluate the genomic correlation between the hypervirulent strains of this study and those isolated from clinical cases of listeriosis occurred in Italy.

Further WGS-based studies are needed, including intensive environmental sampling for *L. monocytogenes* at the slaughterhouse and analysis of finished products are needed, in order to deepen our knowledge on the genetic population of *L. monocytogenes* in the poultry chain, identify persistence niches and understand the dynamics of food contamination. Such data would support Food Business Operators to implement effective mitigation strategies to prevent and/or minimize poultry meat contamination.

## Figures and Tables

**Table 1:** MLST analysis: number of strains belonging to different CCs and STs isolated in each slaughterhouse.

Slaughterhouse	CC1-ST1	CC6-ST6	CC9-ST9	CC121-ST121	CC193-ST193	Total
M1	26	28	6	10	1	71
M2	0	0	48	3	0	51
Total	26	28	54	13	1	122

**Table 2:** Main clusters detected, CC, number of isolates, slaughterhouse, timeframe, ranges of allelic distances and SNPs.

CC	Cluster	N° of Strains	Slaughterhouse	Timeframe	AD	SNPs
CC1	I	26	M1	October 2016–January 2018	0–7	4–10
CC6	I	16	M1	May 2016–January 2018	0–7	1–17
	II	4	M1	July 2016–January 2018	1–7	6–12
	III	3	M1	July 2016–January 2018	0–1	1–3
	IV	2	M1	April–October 2017	1	0
	V	2	M1	April 2017	1	2
CC9	I	30	M2	September–December 2017	0–7	0–16
	II	15	M2	September–December 2017	0–5	1–20
	III	2	M2	September 2017	1	0
	IV	6	M1	April 2017–January 2018	0–1	1–6
CC121	I	4	M1	May–October 2017	0–7	1–19
	II	6	M1	April–October 2017	0–1	0–5
	III	2	M2	September–December 2017	1	1

CC—Clonal Complex; AD—allelic distance.

**Table 3:** Relevant features for virulence, stress response and environmental adaptation.

Main function		Gene/Island	Clonal Complex
Virulence		LIPI-3	CC1, CC6
		*gltA-gltB*	CC1, CC6
		*aut_IVb*	CC1, CC6
		*lapB*	CC1, CC6, CC9, CC121
		*ami*	CC9, CC121, CC193
		*tagB*	CC9, CC121, CC193
		*inlF*	CC9, CC193
		*inlG*	CC9
		*inlL*	CC9
Metal resistance	Cadmium	*cadA, cadC*	CC1
	Arsenic	*arsB, arsC*	CC1, CC6, CC9, CC121, CC193
		*arsA, arsD*	CC9
	Copper	*CsoR-copA-copZ*	CC1, CC6, CC9, CC121, CC193
		*copB*	CC9, CC193
Stress response	Acid Tolerance	*gadB, gadC*	CC1, CC6, CC9, CC121, CC193
		SSI-1	CC9
	Alkali response	*mrpA, mrpB, mrpC, mrpE, mrpF*	CC1, CC6, CC9, CC121, CC193
		SSI-2	CC121
	Low pH, high salt concentration, refrigeration	SSI-1	CC9
	Oxidative stress	SSI-2	CC121
	Cold-shock	*cspD*	CC1, CC6, CC9, CC121, CC193
Biocides resistance		*EmrB, mepA, bmrA, bmr3, norM*	CC1, CC6, CC9, CC121, CC193
	QACs	*Tn6188_qacH*	CC121
Biofilm production		*Lmo0673, lmo2504, luxS, recO*	CC1, CC6, CC9, CC121, CC193
		*inlL*	CC9
		*PMSC inlA*	CC9, CC121, CC193

QACs—quaternary ammonium compounds.

## Data Availability

Sequencing data are available within the NCBI BioProject under accession PRJNA947067.

## Supplementary Materials

The following supporting information can be downloaded at: https://www.mdpi.com/article/10.3390/microorganisms11061543/s1, Table S1: Metadata of all the 122 *L. monocytogenes* used in this study; Table S2: SNPs distance matrix.
